# Posterior urethral valve associated with orthotopic ureterocele

**DOI:** 10.4103/0970-1591.44272

**Published:** 2008

**Authors:** Arun Chawla, Sreedhar Reddy, K. Natarajan, Joseph Thomas, K. Sasidharan

**Affiliations:** Division of Urology, Kasturba Medical College, Manipal, India

**Keywords:** Orthotopic ureterocele, posterior urethral valves

## Abstract

Symptomatic presentation of orthotopic ureterocele in infancy is very rare and its association with posterior urethral valves has not been reported till date. The first such case and a review of the literature on anomalies in association with posterior urethral valves is presented.

## INTRODUCTION

Posterior urethral valves are mostly encountered in isolation and rarely form a part of a conglomerate of congenital lesions. Clinical expression of orthotopic ureterocele in infancy is extremely rare. The simultaneous occurrence of orthotopic ureterocele and posterior urethral valves has no embryological linkage and should be construed as unrelated events. The case of orthotopic ureterocele reported herein evokes interest from the point of view of its hitherto unreported association with posterior urethral valves and its presentation in infancy.

## CASE REPORT

**Figure 1:**
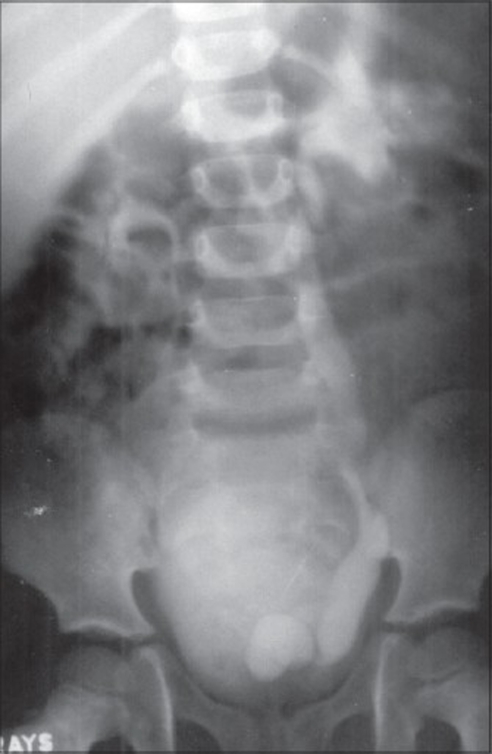
IVU showing left-sided hydroureteronephrosis and orthotopic ureterocele

**Figure 2:**
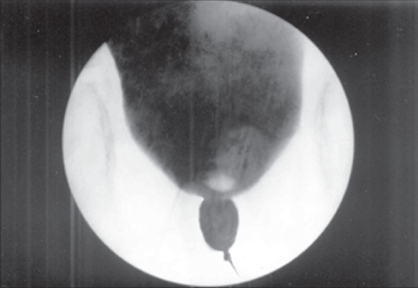
Voiding cystourethrogram showing dilated posterior urethra secondary to posterior urethral valves

**Figure 3:**
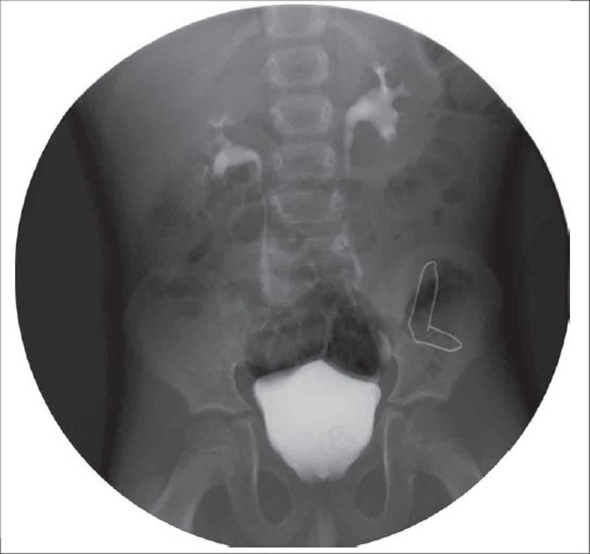
IVU (three months after procedure) showing resolution of left hydronephrosis

**Figure 4:**
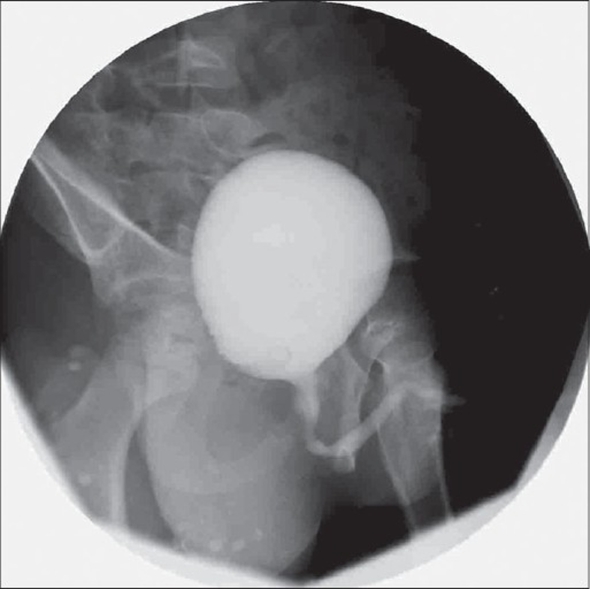
MCU (three months after procedure) showing posterior urethral configuration

A three-year-old male child presented with obstructive voiding symptoms since birth. Initial assessment revealed a well-preserved infant with normal milestones. All routine laboratory investigations were unremarkable. Further evaluation of the child with Intravenous urogram(IVU) disclosed a left-sided orthotopic ureterocele and consequent ipsilateral hydroureteronephrosis [Figure 1]. A voiding cystourethrogram demonstrated absence of vesicoureteric reflux and a distinctly dilated posterior urethra characteristic of posterior urethral valves [Figure 2]. Cystourethroscopy readily displayed Type I posterior urethral valves, which were fulgurated and subsequently the orthotopic ureterocele was decompressed through a transverse incision centering the stenotic ureteric orifice. The post operative period was uneventful. IVU and MCU done at the end of 3 months showed restoration of normal voiding and drainage of left renal unit.[Figures 3, 4].

## DISCUSSION

In clinical practice posterior urethral valves are mostly encountered in isolation and they rarely form a part of a conglomerate of congenital lesions. Statistically, cryptorchidism forms the most common associated congenital anomaly with posterior urethral valves; Kruger *et al.*, in a review of 207 male children with posterior urethral valves discerned an overall incidence of cryptorchidism of 12 per cent.[1] Unicaliceal kidney, partial urethral duplication, and scaphoid megalourethra have been described as rare associated congenital anomalies with posterior urethral valves[2–4]

The case of orthotopic ureterocele reported herein evokes interest from the point of view of its hitherto unreported association with posterior urethral valves and its presentation in infancy.

Clinical expression of orthotopic ureterocele in infancy is extremely rare and the majority of symptomatic ureterocele occur in ectopic ureters subtending upper moieties of totally duplicated systems. The simultaneous occurrence of orthotopic ureterocele and posterior urethral valves in our case has no embryological linkage and should be construed as unrelated events. The burgeoning of orthotopic ureterocele to conspicuous proportions despite high bladder pressure generated by posterior urethral valves is noteworthy. Voiding disability is a common symptom of both ureterocele and posterior urethral valves and hence a micturating cystourethrogram to highlight unsuspected posterior urethral valves as exemplified by this case report is indicated.
